# A 16-year old male with cortical blindness and focal motor seizures

**DOI:** 10.4103/0972-2327.70887

**Published:** 2010

**Authors:** S. M. Katrak, A. Mahadevan, Arun B. Taly, S. Sinha, S. K. Shankar

**Affiliations:** Department of Neurology, Jaslok Hospital and Research Centre, Mumbai, India; 1 Department of Neuropathology, National Institute of Mental Health and Neurosciences, Bangalore-560 029, India; 2 Department of Neurology, National Institute of Mental Health and Neurosciences, Bangalore-560 029, India

## Presentation of a Case (Arun B. Taly, S. Sinha)

A 16-year-old boy was brought by an non-governmental organization (NGO) on 26th November 2007 with a history of (a) progressive bilateral painless visual loss since 11th November 2007, initially involving the right eye followed a few days later by the left eye that progressed to complete blindness, and (b) recurrent and frequent partial motor seizures involving both eyelids and left face and upper limb without secondary generalization from 18th November 2007. There was no history of other focal neurological deficits, raised intracranial pressure, sphincter disturbances, fever, and systemic or local eye disease.

In March 2007, he was found to have cervical lymphadenopathy, for which he received empirical anti-tuberculous therapy for 5 months. However, the patient was not compliant and did not have any medical records for review. He hailed from a village and had recently come to Bangalore for a better livelihood.

On examination, he had pigmented nails and multiple hyperpigmented, non itchy and dry skin rashes over both legs and forearms. He had anemia and bilateral posterior cervical matted, mobile, and nontender lymphadenopathy. There was no evidence of hepatosplenomegaly. Neurologically he was alert, cooperative, conscious, and oriented. There was no perception of light, the pupils were bilaterally equal, reacting to light, and the optic fundi were normal. Eye movements were mildly restricted in all directions. There were frequent partial seizures involving eyelids and occasionally spreading to the left side of face and upper extremity. No other motor or sensory deficits were noted. Muscle stretch reflexes were brisk, and plantar response was flexor bilaterally.

After admission, seizures remained poorly controlled in spite of being on multiple anti-epileptic drugs (AEDs). During his hospital stay, postural instability and mild ataxia of gait was noted. Examination of other systems was unremarkable.

## Course in the Hospital

He received anti-tubercular drugs (rifampicin, isoniazid, pyrazinamide, and ethambutol), parenteral sodium valproate, phenytoin, phenobarbitone, and preterminally levetiracetam in appropriate doses, pentoxyphylline, and systemic antibiotic (ceftriaxone). However, he gradually deteriorated and the seizures remained poorly controlled in spite of multiple AEDs. He succumbed to his illness after 12 days of hospitalization on 8th December 2007.

## Investigations

Hemogram revealed Hemoglobin of 9.9g/dL, total leukocyte count 6900/cmm (neutrophils, 42%; lymphocytes, 42%; eosinophils, 8%; monocytes, 8%), ESR 86 mm/h Westergren, PCV 34.4%; MCV 75.3 fl; RBC count 4.57 million/cmm; and platelet counts of 3,06,000/cmm. Peripheral smear revealed normocytic to microcytic hypochromic anemia and mild eosinophilia. Urinalysis was normal, and culture did not yield any growth. Serum biochemistry results were as follows: Glucose 78 mg/dL; Urea 28 mg/dL; Creatinine 0.8 mg/dL; Calcium 8.9 mg/dL; Total Bilirubin 0.5 mg/dL; Alkaline Phosphatase 152 U/L, SGOT 26 U/L; Na+ 129 mEq/L; K+ 4.2 mEq/L; blood cultures did not reveal any growth. Mantoux test was negative. Lumbar cerebrospinal fluid (CSF) was clear, colorless, and revealed four lymphocytes per cmm, 30 mg% protein, and 67 mg% glucose. Tests for antimycobacterial antibody were negative.

**Figure 1:**
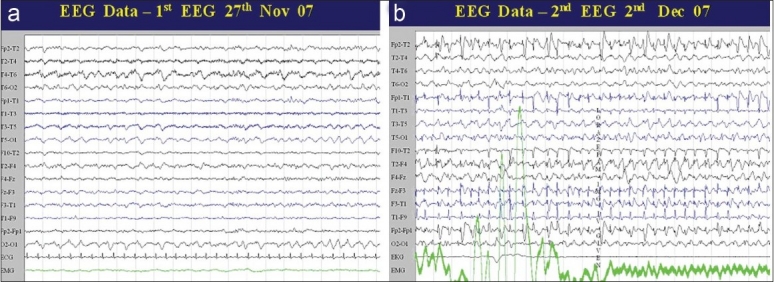
(a) Bilateral posterior slowing right more than left. Absence of α activity and periodic complexes. (b) More diffuse slowing, with deterioration in sensorium. Generalised epileptic activity with partial response to IV lorazepam

Electroencephalography (EEG) was carried out twice (27^th^ November 2007 and 2^nd^ December 2007) [Figure 1].

### Imaging

**Figure 2:**
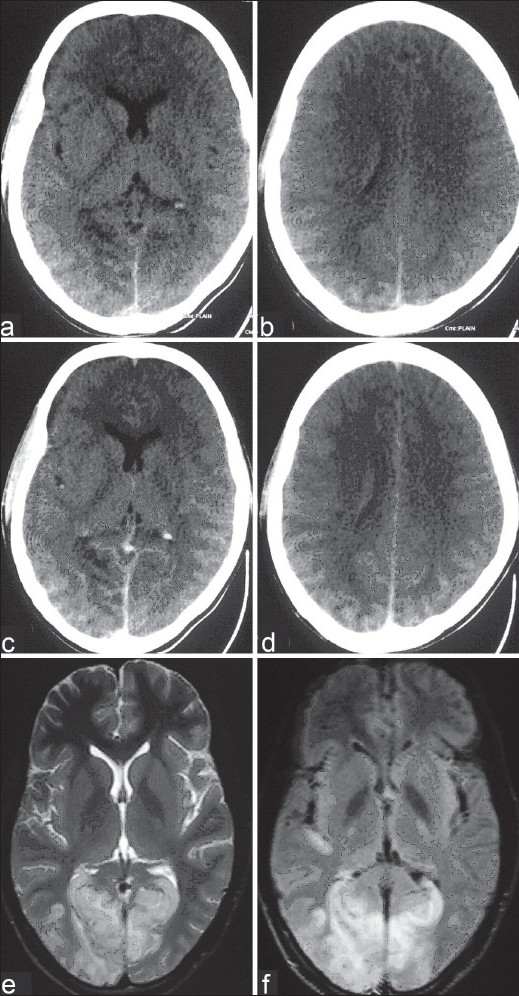
(a–e) : CT scan (2nd December 2007) showing ill-defined bilateral right more than left occipitoparietal interdigitating hypodense lesions with effacement of adjacent sulci and no postcontrast enhancement. (e, f) MRI (29th November 2007) shows bilateral right more than left occipitoparietal T2W and FLAIR hyperintense lesions predominantly involving the white matter but also the gray matter in the right occipital area

Chest X-ray was normal. Cranial CT scan was carried out on the 26^th^ November and again on 2^nd^ December 2007 and an MRI of the brain was performed on 29^th^ Novemmber 2007 [Figure 2].

## Clinical Discussant (S. M. Katrak)

The crux of this patient’s overall illness is a fatal neurological disease associated with cortical blindness, focal motor seizures and mild ataxia associated with pigmented nails, multiple hyperpigmented nonitchy and dry skin rashes over both legs and forearms, anemia, and bilateral posterior cervical matted mobile and nontender lymphadenopathy.

As far as the neurological problem is concerned, we are dealing with a 16-year-old male from a rural background with an acute to subacute onset of an illness without fever, headache, or vomiting but associated with cortical blindness, left focal motor seizures progressing to epilepsia partialis continua (EPC), mild ataxia, and a fulminant progression to death in 4 weeks from the onset of illness.

The differential diagnosis, I have to offer in order of preference are listed in Table 1. which I will discuss individually and give a clinical rating to each of these diseases as (1) highly likely, (2) less likely, or (3) highly unlikely.

**Table 1:** List of differential diagnosis

Acute fulminant subacute sclerosing panencephalitis (SSPE)
Subacute measles encephalitis (SME)/measles inclusion body encephalitis (MIBE)
Acute disseminated encephalomyelitis (ADEM)
Angiotropic B-cell lymphoma
Progressive multifocal leucoencephalopathy (PML)
Acute encephalitic syndromes: viral, bacterial, or other pathogens
Heidenhain’s variant of Creutzfeldt–Jacob disease (HvCJD)

### Acute fulminant subacute sclerosing panencephalitis

The symptoms and clinical signs of acute fulminant subacute sclerosing panencephalitis (SSPE) are practically the same as the chronic form in over 50% of cases except for the fact that they are compressed into a very short span of time. In three large Indian series, involving 391 patients, the age range varied from 3 to 28 years with a male preponderance.[1–3] A predominance of patients from a rural background has been noted by Nagaraja and Arunodaya[2] as well as by Parmeshwaran and Radhakrishnan.[4] A presentation of SSPE without fever, headache, or vomiting is well recognized as is cortical blindness as one of the ophthalmic manifestations,[5,6] as the pathology essentially begins in the occipital lobes.[7] Focal motor seizures and mild ataxia are again well documented in SSPE.[8,9] In a study of 118 cases from the Middle East, the duration of the illness varied from less than 3 months to 6 years[10] and in an Indian autopsy series the shortest duration was 15 days.[11,12] Hence, in this patient, SSPE would be highly likely.

### Subacute measles encephalitis / measles inclusion body encephalitis

The measles virus causes four major CNS syndromes:

- *Acute measles encephalitis*: there is recrudescence of fever during convalescence associated with headache, seizures, and impaired mental state.
- *ADEM*: It occurs a fortnight after exposure to measles infection and will be discussed later.
- *SSPE*: It occurs several years later.
- *Subacute measles encephalitis or measles inclusion body encephalitis*: It can occur 1–7 months after exposure. This has a predilection for children or adults with impaired immunity secondary to an underlying disease or immunosuppressant therapy.[13,14] This rare entity is called measles inclusion body encephalitis (MIBE) or subacute measles encephalitis (SME). The disease runs a course of a few days to a few weeks characterized by afebrile focal seizures, altered mentation, and deterioration in the level of consciousness leading to coma and death.[14,15] The seizures are often severe and takes the form of epilepsia partialis continua and are refractory to anticonvulsant therapy.[14,15] The cerebrospinal fluid may show no abnormalities and anti-measles antibodies are not found, thereby distinguishing this entity from acute fulminant SSPE. In a review of the literature, “visual symptoms/cortical blindness” was detected in 5 out of 33 patients (15%)[13] and “signs of encephalitis were heavily concentrated in the temporal and occipital lobes” in an autopsy proven case.[16] The prognosis is poor with 76% mortality, and all survivors had severe neurological sequelae.[13] This clinical description fits in with the symptoms, signs, and prognosis of the present case. I would rate this clinical entity as highly likely provided that the patient was immunocompromised.

### Acute disseminated encephalomyelitis

Acute disseminated encephalomyelitis (ADEM) may be caused by a host of viral and nonviral pathogens. It usually occurs 2–30 days after a preceding infection but in 33% of children and 50% of adults, no clear preceding infection is elicited.[17] Fever and headache are not invariably present at the onset and are documented in only 43–52% and 45–58%, respectively.[17] The neurological manifestations are dependant on the involved area and may produce cortical blindness. Generalized seizures are reported in 50% of patients with postmeasles ADEM, but persistent focal motor seizures are not very common.[17] Mild ataxia may be due to the involvement of the brainstem or cerebellum, which are common sites of involvement. The prognosis is dependent on the length of coma[11] and mortality of 10–14% has been reported in different series.[14] However, a short fulminant course of a few weeks is rare. Many of the clinical features in our patient cannot be explained by ADEM alone namely, absence of fever or headache, intractable focal motor seizures, cortical blindness at onset and fulminant course. Hence, my clinical rating is—less likely.

### Angiotropic B-cell lymphoma

Angiotropic B-cell lymphoma (AL) is also called malignant angioendotheliomatosis and is characterized by occlusion of arterioles, capillaries, and venules throughout the body by malignant lymphomatous cells. A vast majority (75–85%) have CNS involvement, skin involvement is also recognized, but the hemopoeitic organs and lymph nodes are usually spared.[18] The clinical course is usually fatal in a few weeks to several months.[18] It is for this reason that AL was considered in this case.

An acute to subacute onset is well recognized as is the absence of headaches.[19] However, in a case series of this rare condition, five out of eight patients had fever.[19] Cortical blindness has not been reported in a review of more than 200 reported cases. Nevertheless, the same review reports that one out of the four most frequently cited syndromes is “multifocal cerebrovascular events.”[18] Hence, cortical blindness and mild ataxia still remain a theoretical possibility. Focal seizures have been reported in five out of the eight patients in Beristain’s series.[19] The main feature against this diagnosis in the present case is the age at onset. The reported mean age at onset was 61 years with a range of 41–79 years.[18] Of all the cases of this rare entity, only two cases have been reported in patients below the age of 12 years and one case in a stillborn infant.[18] Hence apart from the fulminant course, the other features in this case do not fit with a diagnosis of AL. My clinical rating would be—less likely.

### Progressive multifocal leucoencephalopathy

Progressive multifocal leucoencephalopathy (PML) is an infection caused by the JC virus. The infection is acquired during childhood but is usually asymptomatic and causes no significant illness. With any form of impaired cellular immunity such as HIV infection, Hodgkin’s lymphoma, non-Hodgkin’s lymphoma, chronic lymphatic leukemia, and rarely tuberculosis, the latent JC virus gets reactivated to cause disease.

The clinical features of PML have been well characterized. Young age at onset is extremely rare and occurrence at 16 years is the earliest recorded. The symptoms are usually insidious, involving the CNS over several weeks. The patients are afebrile, but headaches have been described in 16–32%,[20] and cortical blindness or visual deficits in 19–26% of patients at the onset.[20,21] Focal motor seizures are rare but ataxia is well recognized. The clinical progression to death is over several months and a fulminant course over several weeks is rare.[20,21] In view of the above discussion, I feel that PML is less likely.

### Acute encephalitic syndromes

The clinical hallmarks of acute encephalitic syndromes (AES) are fever, headache, vomiting, and convulsions. In this patient, the first three symptoms are conspicuous by their absence. Although convulsions are a hallmark of AES, the fact that they remain focal motor seizures throughout the course of his illness is very unusual. All the other clinical features may occur in a given case. In view of the absence of fever, headache, vomiting, and the fact that the convulsions remained focal throughout, I consider AES highly unlikely.

### Heidenhain’s variant of Creutzfeld–Jacob disease

In view of the history of cortical blindness, I would briefly like to review Heidenhain’s variant of Creutzfeld–Jacob disease (HvCJD). In a review of 169 neuropathologically confirmed cases of CJD, 34 patients (20%) had HvCJD.[19] The median age at onset was 65.7 years (range, 52–76 years). The onset was insidious without fever, headache, or vomiting. At the onset, clinical features were of visual symptoms such as deterioration of vision, field of vision restriction, optical hallucinations, metamorphopsia, and micropsia have been described. These features progress and in the advanced stages the patients may have cortical blindness. Although myoclonus has been described, focal motor seizures are very unusual. The reported mean duration of illness was 5.7 months and significantly shorter than sporadic CJD (7.5 months).[22] In view of the above-described clinical features of HvCJD, my clinical rating would be highly unlikely.

Summarizing, I would conclude that acute fulminant SSPE or SME/MIBE would be highly likely on clinical grounds. ADEM, AL and PML would be less likely and AES and HvCJD highly unlikely.

### Skin rash, pigmented nails, and lymphadenopathy

In the clinical history, it was mentioned that the patient had “pigmented nails and multiple hyperpigmented, nonitchy and dry skin rashes over both legs and forearms.” Acquired hypermelanosis may be due to physical agents, chemicals, drugs, nutritional, metabolic, and endocrine disorders as well as chronic infections, post-inflammatory skin disorders, and malignant melanosis.[23] From this exhaustive list, one could possibly raise a differential diagnosis of either pellagra, B12 deficiency, Addison’s disease, or the disappearing rash of a recent measles infection. It is possible that the patient had a combination of pellagra and B12 deficiency as it is stated in the history, “he hailed from a village and had recently come to Bangalore for a better livelihood.” Without seeing the skin rash, I find it very difficult to comment on the possibility of Addison’s disease or a disappearing measles rash.

Pigmented nails can have multiple causes and from the sparse history provided it is difficult to comment on them. Suffice it to say, that Addison’s disease and vitamin B12 deficiency are also causes of pigmented nails.

"Matted, mobile, and nontender lymphadenopathy" can be caused by either tuberculosis or lymphomatous process. Considering the main differential diagnosis on the analysis of the clinical features, there appears to be no direct relationship of the skin rash and pigmented nails to SSPE or SME. If the skin rash was due to pellagra, it may reflect malnutrition, which in turn may have produced a disorder of cellular immunity thereby indirectly supporting a diagnosis of SME.[24] It would be tempting to associate the lymphadenopathy with angiotropic B cell lymphoma but as already stated this rare condition usually does not affect the hemopoeitic organs or the lymph nodes.[18,19]

### Laboratory data

The analysis of the laboratory data was unremarkable. The hematological investigations reflected anemia and raised ESR. These are nonspecific changes considering the fact that he may have been malnourished. A high ESR is also known to occur in AL. The Mantoux test was negative, the chest X-ray was normal and the blood cultures were negative.

The CSF examination was normal ruling out an acute encephalitic syndrome. A normal CSF would be compatible with the clinical diagnosis of acute fulminant SSPE,[5] SME,[13] ADEM,[17] AL,[19] and PML.[21] Hence on the basis of the laboratory data, an acute encephalitic syndrome can be conclusively excluded.

### Electroencephalography

The first Electroencephalography (EEG) carried out on 27th November 2007 showed bilateral (right more than left) posterior slowing with absence of α activity. There were no periodic complexes. The second EEG done on 2nd December 2007, when his sensorium had deteriorated, showed more slowing and generalized epileptogenic activity which partially responded to intravenous lorazepam [Figure 1a, b]. This pattern would be consistent with an encephalitic disorder, but is not pathognomonic of any specific infection. A similar pattern has been described in acute fulminant SSPE[25,26] and SME/MIBE[13,15] and would be very strongly against a diagnosis of ADEM and PML. Similarly, the absence of any form of periodic complexes would be a strong point against a diagnosis of HvCJD.[22]

### Neuroimaging data

#### CT scan

The plain and postcontrast cranial CT scan carried out on 26th November 2007 revealed nonenhancing bilateral asymmetrical occipitoparietal predominantly white matter hypodense lesions with evidence of some involvement of the gray matter on the right side. There is effacement of the adjacent sulci. The plain CT scan of 2nd December 2007 revealed ill-defined bilateral but asymmetrical occipitoparietal interdigitating hypodense lesions in the white matter with greater effacement of the adjacent sulci. No focal hemorrhage is evident in these lesions [Figures 2a–d].

The initial brain MRI of 29th November 2007 revealed bilateral asymmetrical T2 weighted hyperintense lesions in the occipitoparietal lobes involving the gray and white matter and being more marked on the right side [Figures 2e, f]. There was effacement of adjacent sulci and mild compression of the occipital horns. In addition, a few ill defined T2 weighted hyperintense lesions in the right high parietal parafalcine cortical gray matter and right posterior parietal cortex were observed. These lesions revealed restricted diffusion, hyperintensity, which appears hyperintense on the ADC maps as well.

The abnormalities described above have been reported in acute fulminant SSPE,[5,14,27] SME,[13,16] ADEM,[17] PML,[21] and AL.[18,28] The involvement in HvCJD is essentially cortical[22] and hence on the basis of clinical, EEG and neuroimaging data, this entity can be safely excluded.

### Therapy

The patient received four drug anti-TB therapy, intravenous AEDs in appropriate doses, systemic antibiotics and pentoxyfylline. I have no issues on the therapy given to the patient except to comment on pentoxyfylline. In recent times, it is documented that pentoxyfylline not only has an inhibitory effect on tumor necrosis factor alpha (TNF-α)[29] but also inhibits viral replication.[30,31] Since SSPE and SME/MIBE are disorders of persistent measles virus infection, I wonder whether it was used for its antiviral properties!

## Conclusion

Reviewing the analysis of the clinical, laboratory, EEG, and neuroimaging data, I would like to conclude that if the patient is immunocompetent the most likely diagnosis is acute fulminant SSPE and if he is immunosuppressed, SME/MIBE is considered. Angiotropic lymphoma would be a lower possibility if immunosuppressed. ADEM and PML are very low on my list of differential diagnosis and I exclude acute encephalitic syndromes and HvCJD.

### Questions to clinician

I do have a few questions to ask the clinicians looking after the case.

1. Which NGO brought the patient? There are NGO’s who look after HIV-affected children.
2. What was the diagnosis of the skin rash and pigmented nails?
3. Was an FNAC or biopsy done of the lymph nodes?
4. What was the HIV status of the patient?
5. What were the measles antibody titers in the blood and the CSF?

### Response of the treating physician

Following the above discussion, Dr. Arun B. Taly responded to the questions and also gave their differential diagnosis.

- The NGO that brought the patient to us primarily takes care of street children and not specifically HIV-affected children.
- Skin and nail changes were suspected to be due to immunocompromised state as are observed in HIV.
- No, the lymph nodes were not subjected to FNAC or biopsy.
- Patient was detected positive for HIV.
- Antemortem analysis of CSF and serum did not include antemeasles antibodies test.

### Final clinical diagnosis

As the measles antibody titers were positive in the blood but negative in the CSF and the patient was seropositive for HIV, the diagnosis should be subacute measles encephalitis (SME)/measles inclusion body encephalitis (MIBE).

## Pathology Discussant (A. Mahadevan)

**Figure 3:**
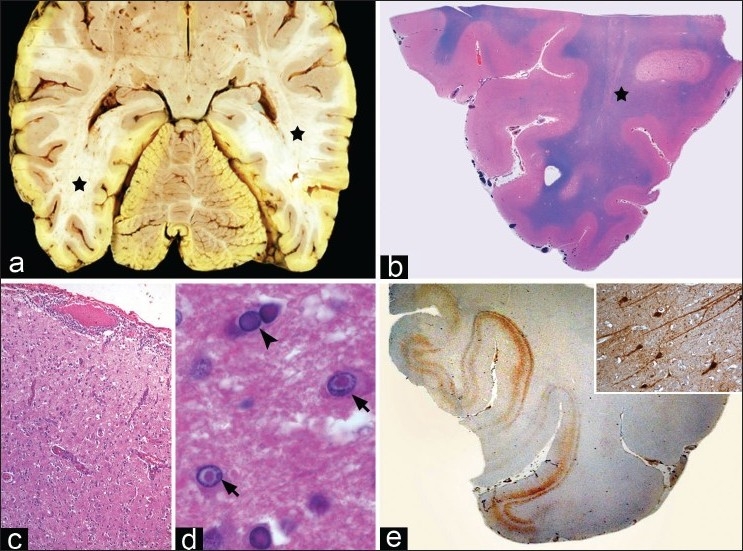
(a) Axial slice showing softening and granularity of optic radiation bilaterally (asterisk, a) with patchy demyelination on Luxol Fast blue (b, asterisk). Occipital cortex (c) showed meningoencephalitis and intranuclear eosinophillic inclusions within neurons (arrows, d) and oligodendroglia (arrow heads, d). Immunolabeling with measles antibody (e) highlights distribution of measles viral antigen involving striate cortex with spread of viral antigen in neuronal soma and dendrites (inset, e). (b: LFB × 8, c: HE × 80, d: HE × 320, e: immunostaining × 8, e, inset: immunostaining × 200).

A complete autopsy was performed following consent from the guardian within 3 h postmortem. The brain was removed through a standard retromastoid bicoronal incision. The brain was diffusely soft, edematous, deeply congested with bilateral cerebellar tonsillar herniation. The occipital poles bilaterally were softened, pale and breaking down. On sectioning, ill-defined zones of granular breakdown was noted along the superior longitudinal fasciculus and the subcortical U fibers in the parietooccipital regions, bilateral medial occipital regions (right more than left) extending along the optic radiation corresponding to the hyperintense lesions noted on neuroimaging [Figure 3a]. The overlying cortical mantle along the right medial occipital cortex appeared irregular and widened. These foci appeared pale with no signs of hemorrhagic discoloration. Horizontal slices through the brain stem and cerebellum appeared grossly normal.

Large sections taken from different neuroanatomical areas were histopathologically evaluated. Evidence of an encephalitic process that varied in extent and severity was prominent. An active florid encephalitic process restricted to cortical ribbon was observed in bilateral medial occipital regions corresponding to hyperintensities seen on MRI [Figure 3c]. A moderately dense meningeal inflammation was overlying the cortical mantle that revealed tissue breakdown and dense inflammatory infiltrates of lymphocytes, histiocytes, and a few plasma cells cuffing thickened vessels in lower layers of cortex. Prominent large hypertrophic reactive astrocytes were seen diffusely distributed throughout the gray matter. Neurons and oligodendrocytes revealed characteristic intranuclear smudgy eosinophillic Cowdry type A inclusions [Figure 3d]. On immunostaining, abundant measles viral antigen was detected within infected neurons within the neuronal nuclei, soma, and dendrites with focal extension into overlying molecular layer particularly in the occipital cortex [Figure 3e]. Oligodendrocytes also contained measles viral antigen albeit less conspicuous than neurons. The underlying white matter showed large zones of demyelination extending from subcortical U fibers to deeper white matter [Figure 3b]. These foci had reactive astrocytosis demonstrated by GFAP immunostains but lacked inflammation. Similar zones of demyelination along with white matter tracts was seen involving right parietooccipital cortex, posterior limb of internal capsule on right, bilateral occipital white matter, and optic radiation and splenium of corpus callosum. Viral inclusions in neuron and glia were less conspicuous, and measles viral antigen detected by immunostaining was less widespread being limited to lower layers of cortical gray in patchy distribution. No viral antigen could be demonstrated in the cerebellum, and brain stem except for a small group of anterior horn cells in cervical segment of the spinal cord. Interestingly, abundant amounts of measles viral antigen was demonstrable in the retinal ganglion and nerve fiber layer as well as plexiform layers of the retina.

Immunostaining for HIV p24 antigen, and opportunistic DNA viruses (CMV, HSV, VZV, and JCV) as well as *Toxoplasma gondii* were negative.

Histological examination of other visceral organs revealed measles-related giant cell pneumonitis in the lung. Measles antigen was also demonstrable in the thymic lymphocytes, spleen and enlarged reactive lymph nodes, suggesting disseminated systemic measles viral infection with measles encephalitis in a HIV-positive immunosuppressed child.

Presence of measles virus in the brain was further confirmed by PCR, and scanning electron microscopic demonstration of parvoviral particles in brain tissue by negative staining (kind courtesy of Dr. Wairagkar, Dr. Atanu Basu, Measles Group, National Institute of Virology, Pune). Molecular genetic analysis excluded vaccine strain and confirmed wild-type virus of D8 genotype. No mutation was demonstrable in M gene of the measles virus. There was no demonstrable measles antibody titers in CSF and efforts to isolate virus from CSF by conventional means was not successful.

**Final diagnosis:** Measles Inclusion Body Encephalitis (MIBE) /subacute measles encephalitis (SME) in an immunocompromised (retroviral positive) child.

## Discussion

### Measles and the central nervous system

Central nervous system involvement in measles though rare, produces a spectrum of syndromes named according to time lapse from the primary infection, with age of occurrence, immune status of host, and neuropathology being distinct [Table 2].

**Table 2:** Clinicopathological differences in measles-related CNS complications

	ADEM	MIBE	SSPE	Fulminant SSPE
Onset after primary infection	Days–weeks	1–7 months	6–10 years	1–6 months
Host	Immunocompetent	Immunosuppressed	Immunocompetent	Immunocompetent
Histology	Inflammation and demyelination, no viral inclusions	Inflammation minimal, viral inclusions++	Inflammation ++, viral inclusions ++	Inflammation ++, viral inclusions few
CSF	Elevated protein No MV antibodies	Normal with no MV antibodies	High titers of MV antibodies	High titers of MV antibodies
EEG	Nonspecific	Nonspecific	Characteristic periodic complexes	Similar to SSPE, can be atypical
MRI	Multifocal, essentially monomorphic signal changes in white and gray matter	Normal at presentation	Characteristic bilateral parietooccipital white matter signal changes among others	Similar to SSPE, can be atypical
Outcome	Monophasic, favorable	Fatal within 1–5 weeks	Fatal within 1–2 years	Fatal within 1–6 months

+, ++, +++ represents density of infl ammation and viral inclusions

### Measles inclusion body encephalitis

MIBS was first recognized by Breitfeld *et al*.[32] in two children with leukemia who developed progressive neurologic disease and died within 6 months following exposure to measles. At autopsy, giant cell pneumonia and acute encephalitis with numerous intranuclear and intracytoplasmic measles viral inclusions were found in the absence of inflammation and giant cells in the brain. By 1993, 31 cases in 16 reports were published and reviewed by Mustafa *et al*.[13] with approximately 12 more cases reported thereafter. All involved children and young adults (up to 21 years) with mean age of 6.1 years at presentation. Acute lymphoblastic lymphoma was the underlying disease in 70% followed by neuroblastoma in 9%. A history of measles was available in 23 cases (70%) (classical in 16 and “non classic” in seven) preceding neurological symptoms by 1–7 months. Most common clinical presentation was seizures refractory to treatment (97%), altered sensorium followed by hemiparesis/hemiplegia (24%), and visual symptoms/cortical blindness (15%). The majority (85%) succumbed within 8 days to 5 weeks from the onset of symptoms. Seizures were most often in the form of epilepsia partialis continua. Brain tissue examined revealed characteristic intranuclear and intracytoplasmic inclusions in neurons and glial cells with paucity of inflammation. Typical tubular paramyxovirus nuclecapsid was detected on electron microscopic examination but measles viral isolation from brain tissue was possible in only two cases.

Diagnosis is difficult as immunosuppression modifies clinical and laboratory features. Skin rash and fever that marks the onset of immune response to measles is often absent. CSF examination is invariably normal and lacks high levels of measles antibodies that characterizes SSPE, consistent with lack of immune response to the virus. EEG findings at the onset though abnormal are often nonspecific and nondiagnostic. MR imaging can be normal at presentation. Tissue diagnosis by brain biopsy and PCR for viral RNA is the mainstay of diagnosis. Molecular biological studies of viral RNA extracted from brain tissue has shown that mRNA for envelope protein is reduced in amount and in some cases mutations in M protein gene similar to SSPE have been found.

In HIV-infected persons, the most commonly reported complication of measles is giant cell pneumonitis. However, measles-associated neurological disorders in children and young adults have been described, including acute encephalitis,[33–35] subacute encephalitis or MIBE,[13,36,37] subacute sclerosing panencephalitis,[38,39] and myelopathy.[35,40] Approximately, eight cases of HIV-associated MIBE are on record in literature, occurring 1–6 months after measles virus infection.[13,34–37,41–43] Most are children or young adult males (age range, 2–20 years) and diagnosis is most often made at autopsy by immunohistochemical identification of measles inclusions or PCR on brain tissues. All case had fatal outcome. Two had received prior measles vaccination.

More recently, cases following immunization with live attenuated measles viral vaccine and stem cell transplantation have been recorded.[15,44] Molecular analysis assists epidemiological studies as it helps to differentiate vaccine strain from wild-type virus.

No treatment has been found to be effective in MIBE although ribavarin that inhibits viral replication in tissue culture, and has been shown to reduce severity and duration of measles-related complications in the immunocompetent. Essentially, all cases of MIBE are fatal, though sporadic reports of long-term survival following high-dose intrathecal ribavirin are on record,[13] similar to SSPE.[45] Ross *et al*. recorded an HIV-infected hemophiliac with measles pneumonitis and encephalitis who responded to intravenous ribavirin.[33] Adequate measles immunization before onset of immunosuppressive illness prevents cases of MIBE, but its safety in HIV-infected individuals is controversial.[46]

Fulminant SSPE, another rare variant form of SSPE is being increasingly reported with approximately 32 cases on record.[12,47,48] It differs from classical SSPE which is a slowly progressive disorder, by a short and fulminant course, with an interval from time of onset of symptoms to death ranging from 1 to 6 months in contrast to a median of 1.8 years for SSPE. Most are clinically atypical with only 15 of 32 cases showing characteristic clinical and EEG findings suggestive of SSPE. Rapid progression of the disease from Stage I–IV is probably responsible for the rarity of occurrence of myoclonus, typical periodic discharges on EEG, and CT/MRI findings. High-titer measles antibodies are found in CSF, but measles viral inclusions in brain is very infrequent making diagnosis difficult histologically.

MIBE or SME is emerging as an opportunistic infection in immunocopromised hosts unable to eliminate measles virus following primary infection. Strain differences in measles virus with emergence of neurovirulent strains consequent to mutations in the gene are considered a possibility, but remains to be elucidated. With increasing pool of patients who are immune compromised, it is likely that this disease will be increasingly recognized. The altered host response resulting in atypical clinical presentations, alterations in electrophysiological, imaging, and laboratory parameters make clinical diagnosis a challenge. Awareness of these clinical variants and high index of clinical suspicion is essential for diagnosis. It is suggested that a history of exposure to measles, or immunization in the previous 7 months should be sought in any immunocompromised child with refractory seizures, even in the absence of fever or skin rash. Brain biopsy, PCR, and molecular genetic analysis in CSF and brain tissue remains the mainstay of diagnosis.
